# Evaluation of adverse events focusing on infection associated with infliximab originator and biosimilar using a spontaneous reporting system database

**DOI:** 10.1186/s40780-019-0149-z

**Published:** 2019-10-07

**Authors:** Iku Niinomi, Keiko Hosohata, Yasuhiro Mori, Yuki Yamaguchi, Tomohito Wakabayashi, Mayako Uchida, Kazunori Iwanaga

**Affiliations:** Education and Research Center for Clinical Pharmacy, Osaka University of Pharmaceutical Sciences, 4-20-1 Nasahara, Takatsuki, Osaka, 569-1094 Japan

**Keywords:** Biosimilar, Infliximab, Adverse drug events, Spontaneous reporting system, Reporting odds ratio (ROR), Japanese Adverse Drug Event Report (JADER) database

## Abstract

**Background:**

Infliximab (IFX) has changed the management of many life-threatening immune-mediated diseases. The high cost of IFX and its patent expiry have led to pharmaceutical companies developing a biosimilar; however, its safety profile remains unknown in the real world. The purpose of this study was to clarify the adverse events associated with IFX originator and its biosimilar using the Japanese Adverse Drug Event Report (JADER) database.

**Methods:**

Adverse event reports submitted to the Pharmaceuticals and Medical Devices Agency between the third quarter of 2014 and the fourth quarter of 2018. We calculated the reporting odds ratio and 95% confidence interval for each adverse event.

**Results:**

We obtained 2771 reports of adverse events associated with IFX originator and 402 reports with IFX biosimilar. Signals were detected for pneumonia, interstitial lung disease, tuberculosis, and sepsis with both IFX originator and its biosimilar, whereas there was no signal for infection with the biosimilar.

**Conclusions:**

The strength of the association between IFX originator and its biosimilar with adverse events is partly different, but reports were quite limited for the biosimilar compared with originator. It is recommended that research be continued in order to accumulate a wide variety of information, and that newly reported data be placed in the multifaceted viewpoints for improvement of care levels.

## Introduction

Infliximab (IFX) is an anti-tumor necrosis factor (TNF)-alpha chimeric monoclonal antibody used in the management of autoimmune inflammatory disorders such as rheumatoid arthritis (RA), psoriasis, Crohn’s disease, and inflammatory bowel disease (IBD). These inflammatory diseases reduce the quality of life of patients [1], so the introduction of IFX has changed the therapeutic approach [2]. Despite its efficacy in biological therapy, IFX as well as other anti-TNFα agents are expensive and have become a burden on pharmacy budgets in most countries, possibly restricting access for many patients [3, 4]. However, many biological products have reached or are close to patent expiration. This has led to the development of biosimilar drugs [5].

Biosimilars are biological medicinal products containing a version of the active substance of an already authorized original biological medicinal product, for which they are required to have similar efficacy, safety and immunogenicity. The similarities between the originator and biosimilar were determined in two phase III clinical trials in patients with RA (PLANETRA) [6] and ankylosing spondylitis (PLANETAS) [7]. However, its safety profile remains unknown in the real-world setting. Especially, the main concern regarding anti-TNF therapy, as well as other biological agents, is the greater susceptibility to infections such as interstitial lung disease, pneumonia, sepsis, and tuberculosis (TB).

Recently, spontaneous reporting systems have been used as a crucial source of post-marketing drug safety surveillance for the detection of adverse drug events [8, 9]. The Japanese Adverse Drug Event Report (JADER) database is a large published database managed by the Pharmaceuticals and Medical Devices Agency (PMDA) for pharmacovigilance [10–12]. The objective of this study was to assess adverse events focusing on infection associated with IFX originator and its biosimilar using the JADER database.

## Methods

Data were extracted from the public release of the JADER database of PMDA, covering the period between the third quarter of 2014 and the fourth quarter of 2018. The main reason for limiting our research to this period was that IFX biosimilar was first launched in November 2014 in Japan. The data structure of JADER consists of 4 data sets: patient demographic information (DEMO), drug information (DRUG), adverse events (REAC), and medical history. Preferred terms (PTs) in the Medical Dictionary for Regulatory Activities (MedDRA) serve as the terminology for registration of adverse events in the REAC table. After we removed duplicated data from each table because the same case report will be received from different sources [13], the DEMO table was linked to the REAC and DRUG tables using the ID number.

The contribution of the medication to adverse events was classified into three categories: “suspected medicine,” “concomitant medicine,” and “interaction,” as described previously. In order to exclude the masking effect, defined as the condition whereby the effect of a given drug-event pair might be hidden by the presence of another product [14], we extracted cases that were classified as “suspected medicine.” Of the IFX biosimilars, “IFX biosimilar 1”, “IFX biosimilar 2”, and “IFX biosimilar 3” were launched in 2014, 2017, and 2018, respectively [15]. In this study, we excluded the reports of “IFX biosimilar 2” (*n* = 5) and “IFX biosimilar 3” (*n* = 9) from analysis since insufficient number of reports were provided.

Next, we calculated the reporting odds ratio (ROR). The ROR is the rate of reporting a specific adverse reaction caused by a particular drug divided by the rate of the same adverse events caused by all other drugs present in the database. A signal was considered to be present when the lower limit of the 95% CI of the ROR was > 1.

In this database, age, height, and weight information are indicated in the form of age in decades, height in centimeter-denominated ranges, and weight in kilogram-denominated ranges. Because these data are not continuous variables, we could not conduct multiple analyses using them. All analyses were performed with JMP Pro 12 (SAS Institute Inc., Cary, NC, USA.).

## Results

The total number of drug and reported adverse event co-occurrences with IFX originator was 2771 (494 different events) and 402 (113 different events) with IFX biosimilar. Of those, infection-related adverse events (Table 1) with IFX originator (657 reports) accounted for 23.7% and those with its biosimilar (88 reports) accounted for 21.9%. Adverse event reports with IFX biosimilar were fewer than with its originator. Among the infection-related adverse events associated with IFX originator, the most common was pneumonia, followed by interstitial lung disease, TB, infection, and sepsis in this order (Table 2). As for those with IFX biosimilar, the most reported adverse event was pneumonia, followed by interstitial lung disease and sepsis.

**Table 1:** Definition of infection of interest. MedDRA, Medical Dictionary for Regulatory Activities; PT, Preferred Term

Event of interest	MedDRA PT^a^
Infection	“Arthritis infective,” “Aspergillus infection,” “Atypical mycobacterial infection,” “Cytomegalovirus infection,” “Epstein-Barr virus infection,” “Fungal infection, Infection,” “Infectious pleural effusion,” “Infective myositis,” “Mycobacterial infection,” “*Mycobacterium avium* complex infection,” “*Mycobacterium marinum* infection,” “Post procedural infection,” “Postoperative wound infection,” “Respiratory tract infection,” “Severe invasive streptococcal infection,” “Staphylococcal infection,” “Streptococcal infection,” and “Urinary tract infection,” and “Wound infection”
Interstitial lung disease	“Interstitial lung disease”
Pneumonia	“Eosinophilic pneumonia,” “Pneumonia,” “Pneumonia influenzal,” “Pneumonia mycoplasmal,” “Pneumonia pneumococcal,” “Pneumonia streptococcal,” “Pneumonia bacterial,” “Organising pneumonia,” “Atypical mycobacterial pneumonia,” and “*Pneumocystis jirovecii* pneumonia”
Sepsis	“Sepsis,” “Septic shock,” and “Listeria sepsis”
Tuberculosis	“Disseminated tuberculosis,” “Intestinal tuberculosis,” “Lymph node tuberculosis,” “Peritoneal tuberculosis,” “Pulmonary tuberculosis,” “Tuberculosis,” and “Tuberculous pleurisy”

**Table 2:** Disproportionality analysis of infection-related adverse events of IFX originator and biosimilar

	IFX originator		IFX biosimilar	
	n	ROR (95% CI)	n	ROR (95% CI)
Pneumonia	230	3.65^a^ (3.19–4.18)	42	4.67^a^ (3.39–6.44)
Interstitial lung disease	148	2.34^a^ (1.98–2.76)	20	2.16^a^ (1.38–3.39)
Tuberculosis	114	26.9^a^ (22.2–32.7)	6	8.88^a^ (3.96–19.9)
Infection	112	3.54^a^ (2.93–4.29)	7	1.48 (0.7–3.13)
Sepsis	53	2.41^a^ (1.83–3.17)	13	4.12^a^ (2.37–7.16)

*CI* confidence interval, *IFX* infliximab, *ROR* reporting odds ratio

^a^ signal detected

Interestingly, IFX biosimilar was no associated with infection, with the number of co-occurrences being only seven. On the other hand, the report of infection was high for IFX originator (*n* = 112), and signal was detected (ROR 3.54, 95%CI 2.93–4.29).

## Discussion

The primary emphasis in biosimilar development is on evaluation of the similarity in physicochemical structure and biological function between the biosimilar and originator biologic. There may be minor differences due to their complex nature and production methods; however, when approved, any variability and differences between the originator and its biosimilar will have been shown not to reduce effectiveness [16]. Indeed, several cohort studies in IBD patients treated with IFX biosimilar showed outcomes comparable to those in patients treated with IFX originator [17, 18]. As for the safety profile, clinical trials are considered to be insufficient for fully evaluating their safety profile due to the limited selection of patients, and so pharmacovigilance such as through the JADER database is considered important.

Our results revealed that signals were detected in pneumonia, interstitial lung disease, TB, and sepsis both with IFX originator and its biosimilar. TB is a serious adverse event accompanying the administration of IFX. TNF-α plays a major role in defence against infection and in the formation and maintenance of granulomas; therefore, treatment with TNF-α inhibitors is recognized as a risk factor for TB [19]. The PLANETRA study [6] and PLANETAS study [20], which were conducted to compare the efficacy and safety of IFX originator and its biosimilar, revealed that the incidences of latent TB were very similar for IFX originator and IFX biosimilar. On the other hand, a prospective and observational cohort study showed that no cases of TB were identified during follow-up in 353 patients with IBD receiving IFX biosimilar therapy [21]. In our results, the association of IFX originator with TB was stronger than that of its biosimilar.

In this study, signal was detected for infection with IFX originator, but not in its biosimilar. There is immunogenicity between IFX originator and its biosimilar. Immunogenicity is associated with a reduced response and other adverse events [20]. The degree of immunogenicity is not the same for all biologics, and only minor differences in the formulation, purity, or packaging of a biological drug can affect the immunogenicity profile. However, the difference in this study is considered to be due to a number of factors other than immunogenicity. Adverse event reports were limited for the biosimilar compared with originator. If the reports increase in the future, the conclusion may change. Further studies are needed.

This pharmacovigilance study using the JADER database has several limitations. First, as in all pharmacovigilance studies, we were unable to calculate the true incidence rates, notably due to: 1) lack of the total number of patients receiving the drugs of interest and 2) under-reporting. Adverse events commonly caused by drugs that are well-known are less likely to be reported. Second, the ROR does not provide a robust indication of the signal strength. In spontaneous reporting systems such as JADER, control populations are not included, so the ROR is different from the “odds ratio” that is commonly used in epidemiological studies. In real terms, the ROR indicates an increased risk of adverse event reporting, and not the risk of adverse events. Finally, the present method did not provide us with detailed clinical information on the patients [22].

## Conclusion

The strength of the association between IFX originator and its biosimilar with adverse events is partly different, but reports were quite limited for the biosimilar compared with originator. It is recommended that research be continued in order to accumulate a wide variety of information, and that newly reported data be placed in the multifaceted viewpoints for improvement of care levels.

## Data Availability

The dataset supporting the conclusions of this article is included within the article.

## Abbreviations

DEMO: Demographic information
DRUG: Drug information
IBD: Inflammatory bowel disease
IFX: Infliximab
JADER: The Japanese Adverse Drug Event Report
MedDRA: Medical Dictionary for Regulatory Activities
PMDA: Pharmaceuticals and Medical Devices Agency
PTs: Preferred terms
RA: Rheumatoid arthritis
REAC: Adverse events
ROR: Reporting odds ratio
TB: Tuberculosis
TNF: Tumor necrosis factor

## References

[1] Feuerstein JD, Cheifetz AS (2017). Crohn disease: epidemiology, diagnosis, and management. Mayo Clin Proc.

[2] Tracey D, Klareskog L, Sasso EH, Salfeld JG, Tak PP (2008). Tumor necrosis factor antagonist mechanisms of action: a comprehensive review. Pharmacol Ther.

[3] Cohen RD, Thomas T (2006). Economics of the use of biologics in the treatment of inflammatory bowel disease. Gastroenterol Clin N Am.

[4] van der Valk ME, Mangen MJ, Leenders M, Dijkstra G, van Bodegraven AA, Fidder HH, de Jong DJ, Pierik M, van der Woude CJ, Romberg-Camps MJ, Clemens CH, Jansen JM, Mahmmod N, van de Meeberg PC, van der Meulen-de Jong AE, Ponsioen CY, Bolwerk CJ, Vermeijden JR, Siersema PD, van Oijen MG, Oldenburg B, group Cs, the Dutch Initiative on C, Colitis (2014). Healthcare costs of inflammatory bowel disease have shifted from hospitalisation and surgery towards anti-TNFalpha therapy: results from the COIN study. Gut.

[5] Zheng MK, Shih DQ, Chen GC (2017). Insights on the use of biosimilars in the treatment of inflammatory bowel disease. World J Gastroenterol.

[6] Yoo DH, Hrycaj P, Miranda P, Ramiterre E, Piotrowski M, Shevchuk S, Kovalenko V, Prodanovic N, Abello-Banfi M, Gutierrez-Urena S, Morales-Olazabal L, Tee M, Jimenez R, Zamani O, Lee SJ, Kim H, Park W, Muller-Ladner U (2013). A randomised, double-blind, parallel-group study to demonstrate equivalence in efficacy and safety of CT-P13 compared with innovator infliximab when coadministered with methotrexate in patients with active rheumatoid arthritis: the PLANETRA study. Ann Rheumatic Dis.

[7] Park W, Hrycaj P, Jeka S, Kovalenko V, Lysenko G, Miranda P, Mikazane H, Gutierrez-Urena S, Lim M, Lee YA, Lee SJ, Kim H, Yoo DH, Braun J (2013). A randomised, double-blind, multicentre, parallel-group, prospective study comparing the pharmacokinetics, safety, and efficacy of CT-P13 and innovator infliximab in patients with ankylosing spondylitis: the PLANETAS study. Ann Rheum Dis.

[8] Mahé Julien, de Campaigno Emilie Patras, Chené Anne-Laure, Montastruc Jean-Louis, Despas Fabien, Jolliet Pascale (2018). Pleural adverse drugs reactions and protein kinase inhibitors: Identification of suspicious targets by disproportionality analysis from VigiBase. British Journal of Clinical Pharmacology.

[9] Mendes D, Alves C, Batel-Marques F (2014). Safety profiles of adalimumab, etanercept and infliximab: a pharmacovigilance study using a measure of disproportionality in a database of spontaneously reported adverse events. J Clin Pharm Ther.

[10] Hosohata K, Inada A, Oyama S, Furushima D, Yamada H, Iwanaga K (2019). Surveillance of drugs that most frequently induce acute kidney injury: a pharmacovigilance approach. J Clin Pharm Ther.

[11] Hosohata K, Matsuoka E, Inada A, Oyama S, Niinomi I, Mori Y, Yamaguchi Y, Uchida M, Iwanaga K (2018). Differential profiles of adverse events associated with mycophenolate mofetil between adult and pediatric renal transplant patients. J Int Med Res.

[12] Hosohata K, Inada A, Oyama S, Niinomi I, Wakabayashi T, Iwanaga K (2019). Adverse cutaneous drug reactions associated with old- and new- generation antiepileptic drugs using the Japanese pharmacovigilance database. Clin Drug Invest.

[13] Bate A, Evans SJ (2009). Quantitative signal detection using spontaneous ADR reporting. Pharmacoepidemiol Drug Saf.

[14] Maignen F, Hauben M, Hung E, Van Holle L, Dogne JM (2014). Assessing the extent and impact of the masking effect of disproportionality analyses on two spontaneous reporting systems databases. Pharmacoepidemiol Drug Saf.

[15] Ishii-Watabe A, Kuwabara T (2019). Biosimilarity assessment of biosimilar therapeutic monoclonal antibodies. Drug Metab Pharmacokinet.

[16] Schreiber S (2015). An update on biosimilar drugs for inflammatory bowel disease. Expert Rev Gastroenterol Hepatol.

[17] Keil R, Wasserbauer M, Zadorova Z, Hajer J, Drastich P, Wohl P, Benes M, Bojkova M, Svoboda P, Konecny M, Falt P, Vanasek T, Pesta M, Pesek F, Bouchner L, Kozeluhova J, Novotny A, Bartuskova L, Spicak J (2016). Clinical monitoring: infliximab biosimilar CT-P13 in the treatment of Crohn's disease and ulcerative colitis. Scand J Gastroenterol.

[18] Farkas K, Rutka M, Golovics PA, Vegh Z, Lovasz BD, Nyari T, Gecse KB, Kolar M, Bortlik M, Duricova D, Machkova N, Hruba V, Lukas M, Mitrova K, Malickova K, Balint A, Nagy F, Bor R, Milassin A, Szepes Z, Palatka K, Lakatos PL, Lukas M, Molnar T (2016). Efficacy of infliximab biosimilar CT-P13 induction therapy on mucosal healing in ulcerative colitis. J Crohn's Colitis..

[19] Sester M, van Leth F, Bruchfeld J, Bumbacea D, Cirillo DM, Dilektasli AG, Dominguez J, Duarte R, Ernst M, Eyuboglu FO, Gerogianni I, Girardi E, Goletti D, Janssens JP, Julander I, Lange B, Latorre I, Losi M, Markova R, Matteelli A, Milburn H, Ravn P, Scholman T, Soccal PM, Straub M, Wagner D, Wolf T, Yalcin A, Lange C (2014). Tbnet Risk assessment of tuberculosis in immunocompromised patients A TBNET study. Am J Respir Crit Care Med.

[20] Allez M, Karmiris K, Louis E, Van Assche G, Ben-Horin S, Klein A, Van der Woude J, Baert F, Eliakim R, Katsanos K, Brynskov J, Steinwurz F, Danese S, Vermeire S, Teillaud JL, Lemann M, Chowers Y (2010). Report of the ECCO pathogenesis workshop on anti-TNF therapy failures in inflammatory bowel diseases: definitions, frequency and pharmacological aspects. J Crohn's Colitis.

[21] Gonczi L, Gecse KB, Vegh Z, Kurti Z, Rutka M, Farkas K, Golovics PA, Lovasz BD, Banai J, Bene L, Gasztonyi B, Kristof T, Lakatos L, Miheller P, Nagy F, Palatka K, Papp M, Patai A, Salamon A, Szamosi T, Szepes Z, Toth GT, Vincze A, Szalay B, Molnar T, Lakatos PL (2017). Long-term efficacy, safety, and immunogenicity of biosimilar infliximab after one year in a prospective Nationwide cohort. Inflamm Bowel Dis.

[22] Franciotta D, Kwan P, Perucca E (2009). Genetic basis for idiosyncratic reactions to antiepileptic drugs. Curr Opin Neurol.

